# Reunion Island, a sentinel territory for antimicrobial-resistant bacteria surveillance in the South-Western Indian Ocean: a retrospective survey using hospitalized patient screening, 2015–2017

**DOI:** 10.1186/s12889-020-09591-8

**Published:** 2020-10-01

**Authors:** Noellie Gay, Nathalie Lugagne, Guillaume Miltgen, Olivier Belmonte, Eric Cardinale

**Affiliations:** 1 UMR Animal Santé Territoires Risque Environnement (CIRAD, INRAe, Univ Montpellier), Montpellier, France; 2 Nosocomial infection Unit, Felix-Guyon University hospital, La Reunion, Saint-Denis, France; 3 Bacteriology laboratory, Felix-Guyon University hospital, La Reunion, Saint-Denis, France; 4 UMR Processus Infectieux en Milieu Insulaire Tropical (CNRS 9192, INSERM U1187, IRD 249, Univ La Réunion), La Reunion, Saint-Denis, France; 5 Health Monitoring Unit, Indian Ocean Commission, Port-Louis, Mauritius

**Keywords:** Epidemiology, Hospital, Indian Ocean, Multidrug resistance, Screening, Colonization

## Abstract

**Background:**

In 2015, antimicrobial resistance was identified as a public health priority for the South-Western Indian Ocean (SWIO) (i.e. Comoros, Madagascar, Mauritius, Mayotte (France), Reunion Island (France), and Seychelles). However, in 2020, colonization rates of antimicrobial-resistant bacteria (ARB) in human populations on most islands in SWIO were still not known and neither hospital nor community colonization rates had been estimated. The aim of this study was to estimate the prevalence of colonization of six ARB groups in hospitalized patients residing in the SWIO territories. The six groups comprise extended-spectrum betalactamase producing Enterobacteriaceae (ESBL-E), carbapenem-resistant Enterobacteriaceae (CRE), methicillin-resistant *Staphylococcus aureus* (MRSA), vancomycin-resistant enterococci (VRE), and both ceftazidime and/or imipenem-resistant *Acinetobacter* spp. (ACB), and ceftazidime and/or imipenem-resistant *Pseudomonas* spp. (PSA)).

**Methods:**

Based on comprehensive hospital laboratory ARB screening data, we provide the first estimation of ARB colonization rates in hospitalized patients residing in SWIO (2015–2017). Using ARB colonization rates in Reunion Island (France) as the reference for estimating odds ratio, we identified at risk patients based on their territory of residence.

**Results:**

The survey pointed to significantly higher overall ARB colonization rates in patients from Comoros, Madagascar, Mayotte, and Seychelles compared to Reunion Island as the reference. Extended-spectrum betalactamase producing Enterobacteriaceae was found to be the most common ARB group colonizing patients from SWIO territories. The highest MRSA colonization rates were observed in patients from Mayotte and Seychelles. Colonization by carbapenem-resistant Enterobacteriaceae (CRE) was highest in patients from Mauritius.

**Conclusion:**

These results identify high ARB colonization rates in hospitalized patients from SWIO territories that require further investigation, particularly CRE in Mauritius and MRSA in Seychelles and Mayotte. This study is the first step toward the implementation of a broader regional ARB surveillance system.

## Background

An increase in the prevalence of antimicrobial-resistant bacteria (ARB) was observed in Reunion Island (France) between 1997 and 2007 [1]. Epidemiological surveillance in hospitals in In 2015 Reunion Island pointed to trends in the incidence of extended-spectrum betalactamase producing Enterobacteriaceae (ESBL-E) similar to Occitanian Region in mainland France and a lower incidence for methicillin-resistant *Staphylococcus aureus* (MRSA) [2].

The South-Western Indian Ocean (SWIO) region contains the islands of the Union of Comoros, Madagascar, Mauritius, Mayotte (France), Reunion Island (France), and Seychelles. Based on a review of the literature, ESBL-E and carbapenem-resistant Enterobacteriaceae (CRE) were identified as a main public and veterinary health issue for SWIO [3]. Antimicrobial resistance has been considered a public health priority in the region since 2015. However, the absence of an ARB surveillance network in most SWIO territories and rare publications on the topic prevented researchers from identifying the most affected islands and the implementation of targeted action plans.

Felix-Guyon University hospital in Reunion Island is well suited for medical evacuations and receives most patients evacuated from other islands of SWIO. Since 2015, an ARB screening strategy has been in place for all patients residing abroad who arrive via medical evacuation, or who visited a foreign country in the three preceding months, and/or were hospitalized abroad in the past year. All patients admitted to the intensive care patients in Felix-Guyon hospital are screened to avoid introducing ARB in the unit.

Based on comprehensive hospital laboratory ARB screening data, we estimated the prevalence of colonization by ARB (i.e. ESBL-E, CRE, MRSA, vancomycin-resistant enterococci, and both ceftazidime and/or imipenem-resistant *Acinetobacter* spp. (ACB), and ceftazidime and/or imipenem-resistant *Pseudomonas* spp. (PSA)) of hospitalized patients residing in SWIO. This is the first estimation of the ARB colonization rates in patients from SWIO territories using standard indicators.

## Methods

### Data collection and inclusion criteria

We conducted a retrospective survey of all patients admitted to the Felix-Guyon University hospital, which is the main hospital in Reunion Island, between 2015 and 2017. Only patients who resided in SWIO were included. All the patients were screened for ARB detection (i.e. anal for ESBL-E, CRE, VRE, ACB, PSA, and nasal swabbing for MRSA). For Reunion Island, only patients admitted to the intensive care unit (all patients were screened) were used as the reference to estimate odds ratio.

### Definition of ARB

Bacterial species were routinely identified for all isolates using MALDI-TOF mass spectrometry (Bruker Daltonics, Bremen, Germany). Antimicrobial susceptibility testing was performed using the disc diffusion method according to guidelines published by the Committee on Antimicrobial Susceptibility Testing of the French Society of Microbiology in 2015 [4].

The ARB groups included in the survey were:

(i) *Staphylococcus aureus* resistant to oxacillin was designated as methicillin-resistant *Staphylococcus aureus* (MRSA) according to the French national multidrug-resistant bacteria surveillance network [5];
(ii) Enterobacteriaceae resistant to cefotaxime and/or ceftazidime and/or cefepime were designated as ESBL-E if a synergy between third-generation cephalosporins and clavulanic acid was confirmed by the disc diffusion method according to French recommendations [4]. ESBL-E definition was according to the French national multidrug-resistant bacteria surveillance network [5];
(iii) Enterobacteriaceae resistant to imipenem and/or ertapenem were confirmed for the presence of relevant resistance genes by PCR (X-pert Carba-R, GeneXpert, Cepheid, Sunnyvale, USA) and designated as carbapenem-resistant Enterobacteriaceae (CRE).
(iv) *Enterococcus faecium* resistant to vancomycin and/or teicoplanin and confirmed for the presence of relevant resistance genes by PCR (X-pert vanA/vanB, GeneXpert, Cepheid, Sunnyvale, USA) were designated by vancomycin-resistant enterococci (VRE);
(v) both *Acinetobacter* spp. (ACB) and (vi) *Pseudomonas* spp. (PSA) included in our survey were ceftazidime and/or imipenem resistant.

A patient was considered ARB positive if one or more ARB group was isolated from that patient.

### Statistical analyses

ARB colonization rates in patients were compared based on their territory of residence using a logistic regression analysis with ARB colonization as the dependent variable and the patient’s territory of residence as the explanatory variable. Odds ratio were calculated using patients residing in Reunion Island as the reference and the statistical significance threshold was set at *p*-values < 0.05. Confidence intervals of ARB prevalence were estimated using the Wilson method. All statistical analyses were performed using R software version 3.4.2 [6], the package tydiverse [7], binom [8], and the package finalfit [9].

The study was approved by the French national commission on data protection and liberties (reference 2,210,228 v0, January 10th 2019).

## Results

From January 1st 2015 to December 31st 2017, a total of 4135 hospitalized patients from the SWIO territories were surveyed. A total of 978 out of 4135 (23.7%) patients tested positive for ARB colonization.

**Fig. 1:**
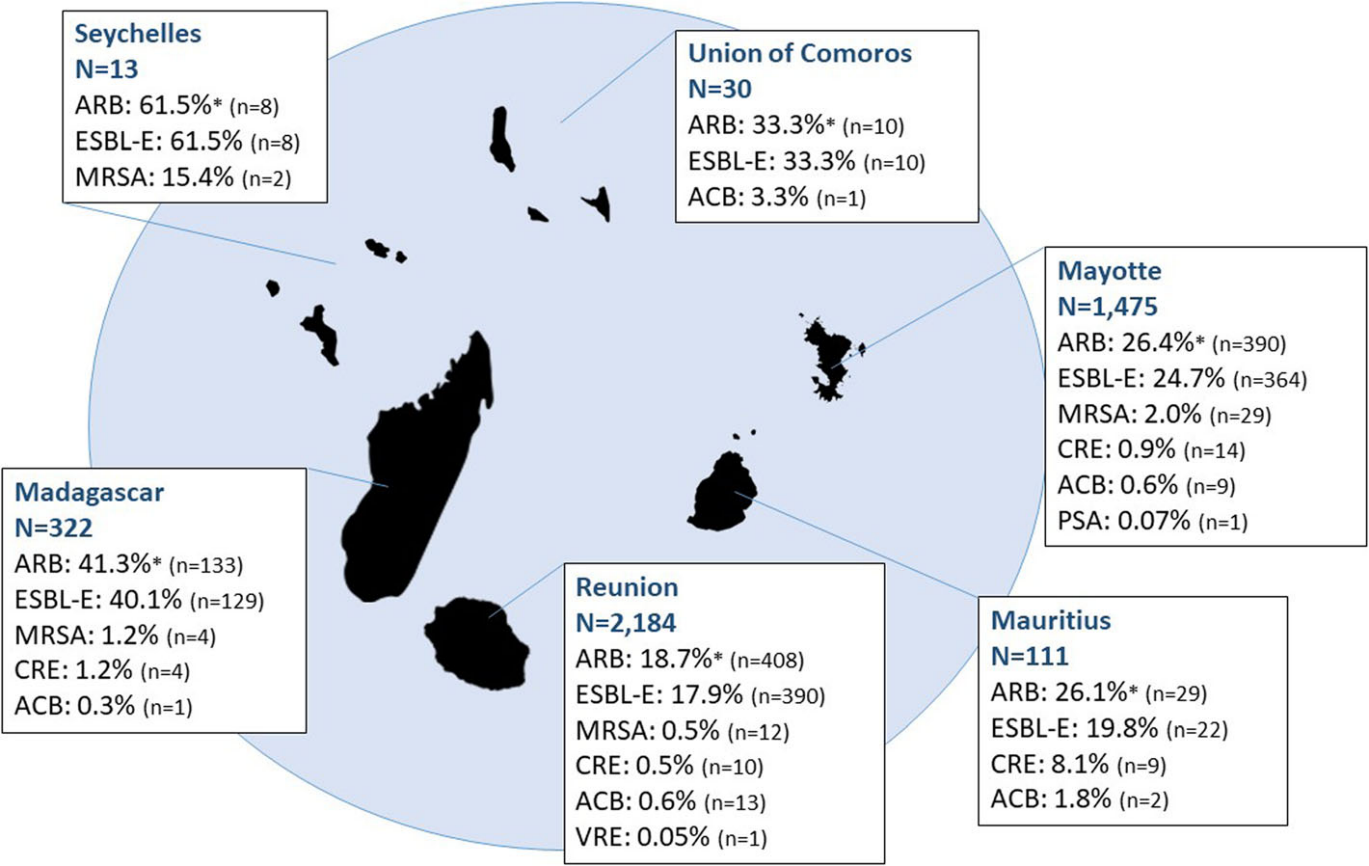
ARB colonization rates according to the patient’s territory of residence in the South-Western Indian Ocean. * An ARB positive patient could be a carrier of more than one ARB group; ESBL-E: extended-spectrum betalactamase producing Enterobacteriaceae; MRSA: methicillin-resistant *Staphylococcus aureus*; CRE: carbapenem-resistant Enterobacteriaceae; ACB: ceftazidime and/or imipenem-resistant *Acinetobacter* spp.; VRE: vancomycin-resistant enterococci.; PSA: ceftazidime and/or imipenem-resistant *Pseudomonas* spp.

The number of hospitalized patients varied between the SWIO territories of residence ranging from 13 patients residing in Seychelles to 2184 patients residing in Reunion Island. The highest ARB colonization rates were observed for patients residing in Seychelles (61.5%) and Madagascar (41.3%) (Fig. 1). A total of 923 out of 978 (94.4%) ARB positive patients were colonized by ESBL-E. ARB colonization rates varied with the patient’s territory of residence.

Overall, ARB colonization rates were significantly higher in Comoros, Madagascar, Mayotte, and Seychelles than in Reunion Island (used as reference); ESBL-E colonization rates followed the same trend (Table 1). MRSA colonization rates were significantly higher in Seychelles and Mayotte hospitalized patients than in patients residing in Reunion Island. CRE colonization rates were significantly higher in patients residing in Mauritius than in patients residing in Reunion Island.

**Table 1:** Comparison of ARB colonization rates according to the patient’s territory of residence (2015–2017) using Reunion Island (France) as reference

	Patients (n)	ARB positive patients		ESBL-E		MRSA		CRE	
		OR (95% CI)	Prevalence (95% CI)	OR (95% CI)	Prevalence (95% CI)	OR (95% CI)	Prevalence (95% CI)	OR (95% CI)	Prevalence (95% CI)
Seychelles	13	**7.0 [2.3–23.2]** ^**^	61.5% [35.5–82.3%]	**7.4 [2.4–24.5]** ^**^	61.5% [35.5–82.3%]	**32.9 [4.8–140.8]** ^**^	15.4% [4.3–42.2%]	_	_
Madagascar	322	**3.1 [2.4–3.9]** ^**^	41.3% [36.1–46.8%]	**3.1 [2.4–3.9]** ^**^	40.1% [34.9–45.5%]	2.3 [0.7–6.6]	1.2% [0.5–3.2%]	2.7 [0.8–8.2]	1.2% [0.5–3.2%]
Comoros	30	**2.2 [1.0–4.6]** ^*^	33.3% [19.2–51.2%]	**2.3 [1.0–4.8]** ^*^	33.3% [19.2–51.2%]	_	_	_	_
Mayotte	1475	**1.6 [1.3–1.8]** ^**^	26.4% [24.3–28.8%]	**1.5 [1.3–1.8]** ^**^	24.7% [22.5–26.9%]	**3.6 [1.9–7.4]** ^**^	2.0% [1.4–2.8%]	2.1 [0.9–4.8]	0.9% [0.6–1.6%]
Mauritius	111	1.5 [1.0–2.4]	26.1% [18.9–35.0%]	1.1 [0.7–1.8]	19.8% [13.5–28.2%]	_	_	**19.2 [7.5–48.6]** ^**^	8.1% [4.3–14.7%]
Reunion	2184	1 (ref)	18.7% [17.1–20.4%]	1 (ref)	17.9% [16.3–19.5%]	1 (ref)	0.5% [0.3–1.0%]	1 (ref)	0.5% [0.2–0.8%]

*ARB* Antimicrobial-resistant bacteria, *ESBL-E* Extended-spectrum betalactamase producing Enterobacteriaceae, *MRSA* Methicillin-resistant *Staphylococcus aureus*, *CRE* Carbapenem-resistant Enterobacteriaceae, *OR* Odds ratio, *CI* Confidence interval, *ref*. reference for odds ratio estimation; ^**^ ≤ 0.001; ^*^ < 0.05; ACB, PSA, and VRE are not presented here as odds ratio were not significant

## Discussion

This study pointed to higher ARB colonization rates in hospitalized patients from Comoros, Madagascar, Mayotte, and Seychelles compared to patients who reside in Reunion Island. ESBL-E colonization was common in all SWIO territories whereas specific epidemiological trends were observed for MRSA and CRE. Higher MRSA colonization rates were reported in patients from Mayotte and Seychelles and a high CRE colonization rate was identified in patients residing in Mauritius (*p* < 0.001). These results are the first step toward a regional hospital-based ARB surveillance system as planned by the Indian Ocean Commission in 2015 [10].

Our survey used the biggest sample of SWIO individuals ever reported in the literature. The study design is based on a convenience sample. Accordingly individuals included in the analysis were not randomly selected, but these data are relevant for the estimation of ARB colonization rates in SWIO hospitals based on the knowledge that i) the patient’s territory of residence is a known risk factor for ARB infection and carriage [11–14] and that ii) individuals included in the study were probably looking for care in Reunion Island after being treated in facilities in the territory in which they reside. Thus, estimated ARB colonization rates should approximate the ARB epidemiological situation in local SWIO hospitals. However, the socio-economic status of patients seeking care on Reunion Island (higher incomes than the local population) may have favor access to healthcare, medicine, and hygiene. Accordingly, estimated ARB colonization rates could underestimate the real colonization rates in local health care settings as poverty is a known risk factor for ARB colonization [15]. Furthermore, using screening data from patients in the intensive care unit in Reunion Island (reference) may also under-estimate the odds ratio. Indeed, the intensive care unit has the highest prevalence of hospital-acquired infections in hospital settings [16]. Finally, the small number of hospitalized patients arriving from Seychelles and Comoros could limit our interpretation.

ESBL-E occurrence in SWIO has already been highlighted as a public health issue both in the community and in hospitals [3] for which our analysis provided quantitative confirmation. For instance, the estimated ESBL-E colonization rate for Madagascar was high (40.1%) which is in accordance with the colonization rates of travelers reported in the literature, which ranged from one in three (33.3%) [17] to four out of seven (57.1%) [18] in 2012–2013, but higher than the 18.5% ESBL-E colonization rate reported in the community in 2013–2014 [19]. A high CRE colonization rate in patients repatriated from Mauritius has already been reported [20]; this may confirm CRE circulation in hospitals in Mauritius and/or in the community. Large fluxes of travelers from India may also contribute to changes in ARB epidemiology in Mauritius [21] as NDM-producing isolates are considered endemic in India [22]. Finally, as ACB and PSA colonization rates were low in SWIO patients, comparisons of the two colonization rates between territories were not possible. Further surveys are needed to confirm the low rates of ACB and PSA colonization observed in this study.

## Conclusion

Our study provides the first estimate of ARB colonization rates in hospitalized patients in the SWIO. Overall high ESBL-E colonization rates were identified in patients from SWIO territories; MRSA and CRE colonization rates were high in certain territories. These results raise awareness on the circulation of specific ARB groups in local SWIO hospitals. This is the first step toward a targeted action plan for the prevention and control of ARB in the region. The results of this study will serve as a basis for the implementation of broader regional surveillance systems.

## Data Availability

The data were obtained from Felix-Guyon hospital laboratory, Saint Denis, Reunion Island. Data were provided for the survey according to the French law (reference 2210228 v0, January 10th 2019) but are not publicly available.

## Abbreviations

ARB: Antimicrobial-resistant bacteria
SWIO: South-Western Indian Ocean
ESBL-E: Extended-spectrum betalactamase producing Enterobacteriaceae
MRSA: Methicillin-resistant *Staphylococcus aureus*
CRE: Carbapenem-resistant Enterobacteriaceae
ACB: Ceftazidime and/or imipenem-resistant *Acinetobacter* spp.
VRE: Vancomycin-resistant enterococci
PSA: Ceftazidime and/or imipenem-resistant *Pseudomonas* spp.
